# Stroke follow-up in primary care: a discourse study on the discharge summary as a tool for knowledge transfer and collaboration

**DOI:** 10.1186/s12913-020-06021-8

**Published:** 2021-01-07

**Authors:** Rune Aakvik Pedersen, Halfdan Petursson, Irene Hetlevik, Henriette Thune

**Affiliations:** 1 Department of Public Health and Nursing, General Practice Research Unit, NTNU, Norwegian University of Science and Technology, Trondheim, Norway; 2 Faculty of Health Sciences, University of Stavanger, Stavanger, Norway

**Keywords:** Stroke, Primary care, Practice guidelines, Fragmented care, Collaboration, Knowledge transfer, Discourse analysis

## Abstract

**Background:**

The acute treatment for stroke takes place in hospitals and in Norway follow-up of stroke survivors residing in the communities largely takes place in general practice. In order to provide continuous post stroke care, these two levels of care must collaborate, and information and knowledge must be transferred between them. The discharge summary, a written report from the hospital, is central to this communication. Norwegian national guidelines for treatment of stroke, issued in 2010, therefore give recommendations on the content of the discharge summaries. One ambition is to achieve collaboration and knowledge transfer, contributing to integration of the health care services. However, studies suggest that adherence to guidelines in general practice is weak, that collaboration within the health care services does not work the way the authorities intend, and that health care services are fragmented.

This study aims to assess to what degree the discharge summaries adhere to the guideline recommendations on content and to what degree they are used as tools for knowledge transfer and collaboration between secondary and primary care.

**Methods:**

The study was an analysis of 54 discharge summaries for home-dwelling stroke patients. The patients had been discharged from two Norwegian local hospitals in 2011 and 2012 and followed up in primary care. We examined whether content was according to guidelines’ recommendations and performed a descriptive and interpretative discourse analysis, using tools adapted from an established integrated approach to discourse analysis.

**Results:**

We found a varying degree of adherence to the different advice for the contents of the discharge summaries. One tendency was clear: topics relevant here and now, i.e. at the hospital, were included, while topics most relevant for the later follow-up in primary care were to a larger degree omitted. In most discharge summaries, we did not find anything indicating that the doctors at the hospital made themselves available for collaboration with primary care after dischargeof the patient.

**Conclusions:**

The discharge summaries did not fulfill their potential to serve as tools for collaboration, knowledge transfer, and guideline implementation. Instead, they may contribute to sustain the gap between hospital medicine and general practice.

## Background

### Stroke follow-up

Stroke is one of the major causes of death and disability worldwide [1, 2]. About 13,000 patients are registered annually with acute stroke as primary or secondary diagnosis in Norwegian hospitals [3]. Most patients are discharged to their own home after the acute hospitalization for stroke [4] and the follow-up of patients residing in the communities takes place in primary care. Norwegian national guidelines for treatment of stroke, issued in 2010, state that general practitioners (GPs) should play a key role in the follow-up of stroke survivors [5]. After the introduction of the Regular General Practitioners System in 2001, all inhabitants in Norway are entitled to a regular general practitioner (RGP). At the time of this study, about 99% of the Norwegian population was registered on RGP’s lists [6].

After a first stroke, people have an increased risk of recurrent strokes [7] which are associated with particularly high mortality [8, 9]. Individualized secondary prevention is meant to reduce mortality and morbidity from stroke and can provide substantial gains [10]. Secondary prevention is part of the follow-up in primary care.

### The discharge summary and collaboration within the health care services

To ensure optimal post stroke care after discharge from hospital, collaboration within the public health care is vital [11]. The guidelines’ developers acknowledge that collaboration and knowledge transfer within the health care service are important factors for the optimal treatment and follow-up of patients. Therefore, they provide specific advice on how collaboration and knowledge transfer should take place, while emphasizing the importance of establishing chains of care that are continuous within and across organizational boundaries in health care [5].

The discharge summary is a written report from the responsible physician at the hospital, generated at the end of the patient’s hospital stay. In Norway, this report is primarily sent to the patient’s RGP. This information transfer is essential to the smooth transition from inpatient to outpatient care [5]. The guideline recommends that discharge summaries describe multidisciplinary assessments and provide specific advice on follow-up, rehabilitation and secondary prevention. Furthermore, ten recommended elements are listed (Table 1).

**Table 1:** Elements of the discharge summary, recommended in the guidelines

• The kind of stroke and its localization in the brain • The cause of the stroke • A short description of the treatment and the diagnostic investigation • Complications (if applicable) • The patient’s level of function on discharge • Prognosis, including prognosis for driver’s license and work • Assessment of the necessity for further diagnostic investigations • Medication at discharge • Further treatment and treatment goals for the blood pressure and blood lipid values • Plans for the follow-up	

By adhering to this part of the guidelines, the discharge summary is meant to serve as a tool for knowledge transfer and collaboration within the health care services. When these recommendations are adhered to, the discharge summary can also serve as a tool for guideline implementation in general practice, e.g. by setting treatment goals for blood pressure and blood lipid values.

However, an increasing amount of evidence suggests that the follow-up in general practice is not in accordance with clinical guidelines [12–16] and that collaboration within the health care services does not necessarily work as intended [17]. Instead, fragmentation and inadequate integration of health services are obstacles in ensuring that the scientific advances in prevention, treatment and rehabilitation are translated into clinical practice [18]. From similar fields of practice, we know that there is a gap between evidence-based recommendations and real-world management [19, 20]. Deficits in communication and information transfer in discharge summaries may adversely affect patient care [21].

It is not known whether the secondary health services use the discharge summaries to provide the GPs with multidisciplinary assessments or specific advice on the follow up, as recommended by the guidelines. Nor is it known whether the discharge summary is used to spread knowledge about the guidelines’ specific recommendations on secondary preventive measures. Furthermore, it is not known to what degree the discharge summary provides an invitation to the recommended collaboration within the health services.

### Discourses

A study of discharge summaries is a study of language in use in a specific setting. Language in use is about saying, but also about being [22] and doing things with words [23]. When saying – or writing – something, we adjust our language to the setting. We enact a practice that belongs to a social group or a culture, and by doing this, we sustain this culture [22]. We take part in the discourse. A discourse can be defined as a cognitive and normative community that is expressed in language [24]. Hence discourses can have different sizes, and there is no end to their numbers [22]. One can talk about a medical discourse, but there are also numerous discourses even within medicine and they are dynamic in time and place. On this basis, it is reasonable to distinguish between the current discourses of specialized medicine or hospital medicine and medicine in general practice or primary care. Hospital medicine and primary care medicine use different diagnostic systems. At the time of this study, hospitals in Norway used International Classification of Diseases and Related Health Problems, 10th revision (ICD 10) [25] while primary care used International Classification of Primary Care, 2nd edition (ICPC-2) [26]. These coding- and classification systems have different backgrounds and different developers. The base version of ICD 10 was published by World Health Organization (WHO) and ICPC-2 was developed by World Organization of Family Doctors (WONCA). The different diagnostic systems are examples of a gap between two discourses, expressed in sign systems and language.

Discourse analyses are qualitative and interpretative analyses. They are concerned with studying language in use. Significance, identities or relationships are examples of what we build in language. It is possible to analyze each of the language constructions by asking predefined questions. As an example, we can ask not just what the author is writing, but also what he or she is trying to do [22]. Discourse analysis consists of a wide range of qualitative analytical approaches from which the researcher must choose one.

### Aims

This study aims to assess possible obstacles to guideline adherence and collaboration within the health care service expressed in the discharge summaries for stroke survivors. More detailed aims were:

- To explore the extent to which the discharge summaries contain the elements recommended by the guidelines.
- To assess to what degree the discharge summaries provided an invitation to a post discharge collaboration.

## Methods

### Design and setting

This study was part of a larger project on stroke follow-up in primary care. In this project we examined adherence to the guidelines [12], and assessed the implications of multimorbidity for the follow-up of patients with stroke in general practice [27]. We found weak adherence to the guidelines in general practice [12] and saw the need to have a closer look at aspects of collaboration.

We included patients treated for stroke in two Norwegian local hospitals in 2011 and 2012. In order to study the follow-up in general practice and the collaboration between hospital and general practitioner, it was essential to identify patients discharged to their own homes. In Norway, these are the patients who are followed-up by their RGPs. While for the previous parts of the project, the material has consisted of the RGPs’ medical records, the material for this study consists of hospital discharge summaries provided by the hospitals. RAaP personally visited each participating RGP clinic in order to collect the material for the first parts of the project, therefore the admission area of the hospitals had to be limited and so the number of hospitals also had to be limited. The two hospitals had a total admission area of about 9500 km^2^ with close to 120,000 inhabitants. The choice of hospitals made it possible to reach any of the RGP clinics within a four-hour drive each way. We considered that longer travel was not feasible. After ethics approval was granted, the hospitals provided lists of patients with discharge diagnoses I63.0 trough I63.9 according to ICD 10, and provided access to the patient files. The patients’ RGPs were identified by The Norwegian Health Economics Administration (Helfo), and invitations to participate in the study were sent to each of these RGPs. The contribution of the RGPs was to facilitate the collection of data in the first part of the project. Only patients living at home and registered with an RGP who accepted participation, were subsequently invited to participate in the study. Written, informed consents were obtained from all participating patients. Participation meant allowing the researchers access to their medical records and nothing else. Patients not able to consent and patients in nursing homes were excluded.

### Discourse analysis

RAaP initially read all the discharge summaries aided by the list of guideline-recommended content categories (Table 1) and registered content recommended in the guidelines. Complications were defined based on the guidelines’ description of common and important complications. Furthermore, the number of discharge summaries that provided multidisciplinary assessments was counted. An operational definitions list is provided in Additional file 1.

In the following discourse analysis, that was performed by RAaP and HT, we used analytical tools adapted from J. P. Gee’s interdisciplinary approach to discourse analysis [22]. He describes a general overarching system with 28 tools for the analysis, while emphasizing the need for adapting tools from any theory to the needs and demands of the individual study and that some tools will be more useful for some kinds of data than for other kinds of data [28]. In practice, we therefore do not use all the tools available but select the ones that appear most suitable for our purpose. We initially conducted an explorative analysis with a wide range of the available tools. Tools that did not provide answers that illuminated our aims were excluded. In this way, we subsequently narrowed down our approach with the tools most suitable for our aims. This approach resulted in the identification of what we at this point in the process regarded the most relevant tools for this material and this study. The selected tools and their operational definitions are presented in Table 2.

**Table 2:** Tools for the discourse analysis adapted from J.P. Gee

Tools	Operational definition
**1) The Subject Tool**	Ask why the authors have chosen the subject/topic and what are they writing about the subject. Ask also if and how they could have added more topics and why they did not.
**2) The Doing and Not Just Saying Tool**	Ask not just what the authors are writing, but also what they are trying to do. Accept that they may be trying to do several things.
**3) The Significance Building Tool**	Ask how language is being used to build up or lessen significance/ importance/ relevance for certain things, but not for others.
**4) The Activities Building Tool**	Ask what activity (practice)/ activities (practices) the text is building/ enacting. What activity/ activities is the text seeking to get others to recognize.
**5) The Identities Building Tool**	Ask what identity or identities the author is enacting or trying to get others to recognize.
**6) The Relationships Building Tool**	Ask how language is being used to build, sustain, or change relationships of various sorts among the authors, other people, groups or institutions.
**7) The Figured Worlds Tool**	Ask what typical figured worlds the words or phrases of the text are assuming and inviting readers to assume. Especially, how is the GPs situation in this figured world?
**8) The Collaboration Tool**	Ask in what way are words and grammatical devices being used to make the text invite to collaboration. Ask also if there are signs of the opposite in the text.
**9) The Patient’s Voice Tool**	Ask if the patient’s voice (questions, utterances, opinions, wishes or preferences) are commented on (other than indirectly in the anamnesis).
**10) The Recipient Tool**	Ask what recipient the author most likely had in mind when writing, based on the subject, contents, words and phrases in the text.

## Results

### Description of the selection and material

A total of 100 RGPs were invited, and 37 agreed to participate. They had 138 patients with stroke as a discharge diagnosis in 2011 or 2012 on their lists. We invited all these 138 patients to participate, and 51 gave their written consent. Age on the date of discharge from hospital varied from 38 to 90 years (mean 68.5 years). Thirty (59%) were male and 21 (41%) were female. The material consisted of 54 discharge summaries. For some of the patients, more than one discharge summary was included. Additional discharge summaries were included for new admissions when stroke was the diagnosis. Three discharge summaries were excluded in the analysis stage of the project. One discharge summary was excluded because the content revealed that the correct diagnosis was transient ischemic attack (TIA) rather than stroke and two were excluded because the content revealed that the patients were treated as outpatients, even though the patients were all registered as inpatients with ischemic stroke in the hospital’s own system.

The patients were treated in two different clinics in different geographic locations. The clinics shared the same administration and offered equal services to stroke patients in their respective geographic areas. They were located in two neighboring towns of equal size in mid-Norway, separated by a distance of about 75 km. In total 28 different physicians were involved in the production of the discharge summaries, that most often were written and signed by a subordinate doctor before they subsequently were approved and counter-signed by a senior doctor; a specialist in neurology or internal medicine. Eight of the texts were written and signed by only one physician, in these cases the physicians were all specialists in neurology or internal medicine.

**Fig. 1:**
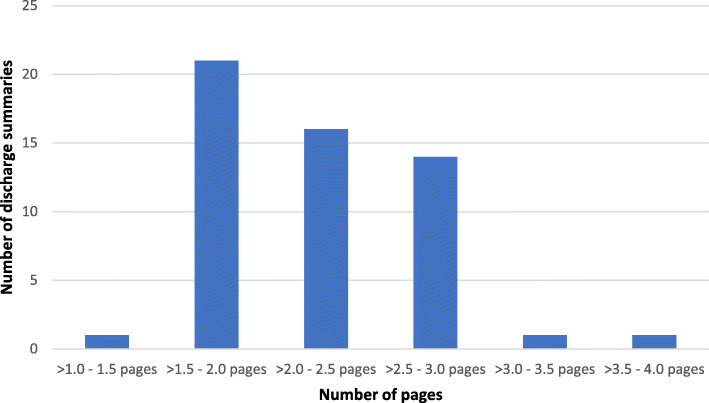
The text lengths of the 54 discharge summaries

Figure 1 illustrates the lengths of the discharge summary texts that varied from approximate one A4 page to four A4 pages.

**Fig. 2:**
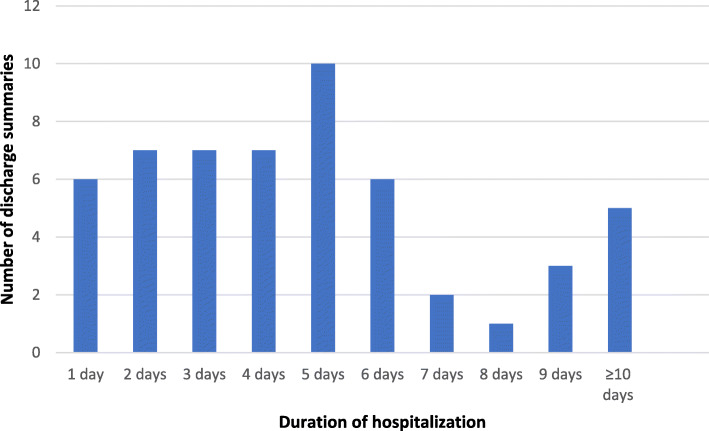
The duration of hospitalization

All discharge summaries included date of admission and date of discharge. The duration of hospitalization varied from 1 day to 20 days and is illustrated in Fig. 2.

### Description of guideline recommended content categories found in the discharge summaries

**Fig. 3:**
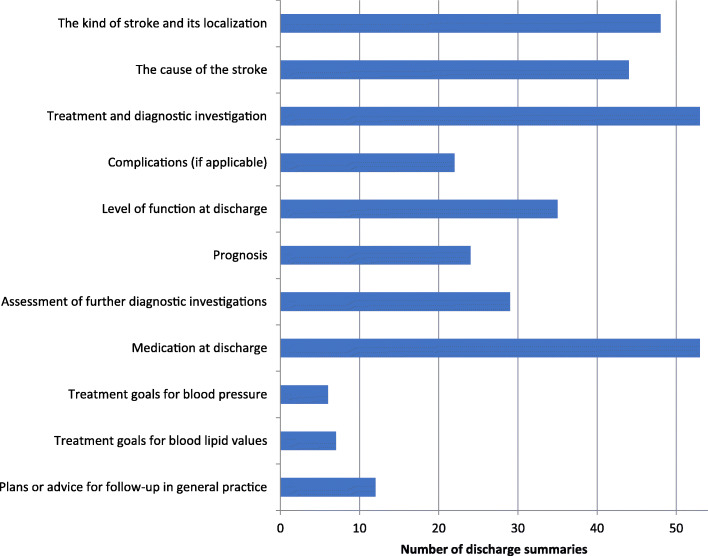
Guideline recommended content in the discharge summaries (*N* = 54)

We found varying degrees of adherence to the different recommendations for content in the discharge summaries (Fig. 3). The discharge summaries often described the kind of stroke and its localization in the brain (87%), the cause of the stroke (80%), medication at discharge (98%) and the treatment and diagnostic investigation (98%).

We found a description of the patient’s level of function on discharge in 34 of the discharge summaries (63%), assessment of the necessity for further diagnostic investigations in 29 (54%), a description of complications in 22 discharge summaries (41%), advice on treatment goals for blood pressure in six discharge summaries (11%) and advice on treatment goals for blood lipid values in seven (13%). Multidisciplinary assessments were provided by 17 (31%) of the discharge summaries.

We used the 10 tools presented in Table 2 on each of the 54 discharge summaries. We present the results divided into those of a descriptive discourse analysis and an interpretative discourse analysis.

### Descriptive discourse analysis

#### Tool 1. The subject tool

When we were working with “The Subject Tool,” we asked what the topic of the text was, if the authors could have made other choices of subject or topics and why they did not. Before presenting the main topic, most discharge summaries provided a brief description of the patients’ background with selected information on past and present illnesses, work, social conditions, family and heredity. Usually this description was kept within a few lines. Some discharge summaries, however, had longer descriptions of the background information. The two longest had respectively 9 and 14 lines devoted to this background information. The shortest descriptions merely stated that the patient was previously healthy before moving on to the next topic:

***Discharge summary 17:*** *“Previously healthy man who (time and date) noticed a numb feeling …*”.

The background description could also be kept short in cases where the patient was suffering from multimorbidity:

***Discharge summary 8:*** *“63-year-old man, with known diabetes mellitus, insulin treated from (year). ACB- operated (year). Woke up (time and date) with numbness and a loss of strength in the right …”.*

The main topic was typically initiated with a brief description of the patients’ symptoms, followed by clinical findings:

***Discharge summary 9:*** *“The patient is admitted with acute difficulties in controlling the right arm, first registered at 06.45 am on the day of admission. On examination a distal loss of strength is registered and loss of tempo in the right arm, dysmetria on finger-nose test. NIHSS score 1p.*

After presenting the clinical findings, most summaries presented findings from supplemental examinations, such as x-rays, CT-scans, MRI-scans, or blood samples. The main topic was then concluded in a chapter on progress, assessment and treatment. In addition to this overarching main theme, sometimes other themes emerged. Examples of such other themes were the patient’s social situation or why a certain treatment was not given.

#### Tool 2. The doing and not just saying tool

When we applied “The Doing and Not Just Saying Tool,” we asked not just what the authors were writing, but also what they were trying to do. Showing that the investigation at the department was finished and complete, and that relevant treatment was initiated was central in all discharge summaries, but the authors often tried to do other things in the same texts. This could be to refer the patient to another department:

***Discharge summary 5:*** *“We find a closer cardiac examination indicated and the discharge summary applies as referral to …”.*

Or to formalize the suspension of a driver’s license:

***Discharge summary 2:*** *“4 weeks suspension of driver’s license after TIA/ stroke without motor/ visual sequela.”*

The texts often made it clear that no further appointments were made. Some were specific and clear about transfer of responsibility to the GP and some established a system for follow-up where the hospital took on a further responsibility for the patient.

Much used, however, were some forms of these short phrases:

*“No further follow-up at our department.”*

or

*“Follow-up by the RGP.”*

#### Tool 3. The significance building tool

This tool is meant to help us identify how words and grammatical devices are used to build up or lessen the importance of certain things. Foregrounded information is given extra importance or relevance in language. We can also build or lessen significance or importance with the words we use. In the discharge summaries, we found that technical investigations were often foregrounded. By the use of specialist language, they were presented in a way that made them seem important:

***Discharge summary 5:*** *“MRI Caput. Sagittal T1, transversal T2, coronal FLAIR, transversal BOLD, and diffusion. Confluent high signal changes around the ventricles, compatible with chronic circulatory changes.”*

However, we also found examples where clinical findings or assessments were made more significant than technical radiological findings. In these cases, the assessments were made by doctors.

***Discharge summary 41:*** *“The patient has clinically had a stroke on an atherosclerotic basis”.*

Assessments from other health care personnel, e.g. physiotherapists were reported summarily and with the use of everyday language:

***Discharge summary 38:*** *“Has received guidance from a physiotherapist who does not see need for physical follow-up beyond self-training.”*

***Discharge summary 29:*** *“She has been assessed by a physiotherapist in the ward, and is considered not to need specialized rehabilitation after discharge.”*

In one of the discharge summaries, we found that language was used in different ways when referring to conversations with respectively a cardiologist and a dietitian:

***Discharge summary 21*** *“Secondary stroke prophylaxis was discussed with a cardiologist”.*

Whereas, from the same discharge summary:

*“the patient ( …*) *got to have a chat with the dietitian...”*

#### Tool 4. Activities building tool

We asked what activity or practice the texts were seeking to get others to recognize. The activities described were the clinical examination at admission, the further diagnostic investigations, clinical assessment, clinical decision-making, and treatment.

#### Tool 5. The identities building tool

When we read the texts aided by “The Identities Building Tool”, the presumed identities enacted might have been the ones of dedicated clinicians at hospitals, carrying out clinically and technically advanced hospital activities. While this often may be the case, we also found examples where the authors enacted other identities:

***Discharge summary 6:*** *“For the sake of order, one reminds that when acute stroke/ TIA is suspected, the patient should be referred to the neurological department for acute assessment.”*

#### Tool 6. The relationships building tool

When we explored how relationships were built, sustained or changed in the discharge summaries, we primarily focused on relationships between doctors in primary and secondary health care and between doctors and patients. We found few indications of relationship building, most often the relationship was changed or ended. Frequently, we found variations of phrases like.

***“****No further appointments in the neurological department.”* (For instance, in **discharge summaries 2, 6, 7, 12,** and **17**).

#### Tool 7. The figured worlds tool

We assessed what typical assumptions that were made in the discharge summaries with a focus on identifying assumptions about further treatment in primary care.

***Discharge summary 1:*** *“The patient has a 4-week suspension of driver’s license after discharge, after which a new assessment must be performed by the GP.”*

***Discharge summary 9:*** *“Requesting the GP to perform follow-up within 4-6 weeks...”*

***Discharge summary 48:*** *“Hb control in about two weeks.”*

#### Tool 8. The collaboration tool

We asked in what way the texts invited to a further collaboration on the patients’ care after discharge from the hospital. In some of the texts, we found advice on what tests should be performed by the GP or a request to the GP to check an abnormal finding made at the hospital.

***Discharge summary 14:*** *“We ask the RGP to control kidney function.”*

***Discharge summary 24:*** *“One asks the RGP to follow up with regular check-ups of blood pressure and lipid status.”*

We did not find direct invitations to further collaboration beyond this in any of the discharge summaries.

#### Tool 9. The Patient’s voice tool

Most texts did not include the patient’s voice. A few included the patients’ views:

***Discharge summary 21:*** *“Patient feels he does not function as normal yet, although he has an apparently good motor function, normal speech and no more reported visual problems.”*

***Discharge summary 44:*** *“The patient was offered a rehabilitation stay at* (place) *but had a strong desire to return home.”*

#### Tool 10. The recipient tool

Sometimes a colleague in another department in secondary care was asked for an assessment or to perform some sort of further treatment or diagnostic investigation. In these cases, the rationale was thoroughly explained.

This was a contrast to cases where the authors concluded that no further follow-up in specialized care was necessary. Then the referral to the colleague in primary health care could be made short:

***Discharge summary 43:*** *“Follow-up of blood pressure by the RGP.”*

***Discharge summary 52:*** “*Further follow-up by the RGP as well as physical therapy training.”*

### Interpretative discourse analysis

The topic (Tool 1) most often seemed to be what has happened there and then; diagnostic investigations and medical treatment. They could also have added other topics, such as collaboration or advice for the follow-up in primary care, but this rarely happens. Considering what the authors were trying to do (Tool 2), an obvious interpretation would be that authors were often trying to do other things than inviting to collaboration or reporting on the elements recommended by the guidelines. A possible interpretation is clearly that they were often trying to end their responsibility for the patients.

Our findings when working with “The Significance Tool” (Tool 3) can be interpreted as gradients in significance. We found traces of a hierarchy. In this hierarchy, technical findings were given high priority, but doctors’ assessment could nevertheless in some cases set the technical findings aside. In some of the texts there was an obvious gradient of significance between different types of health care professionals.

The activities going on (Tool 4) are not necessarily only the activities first assumed. We found a strong focus on what happened there and then in terms of different kinds of examinations, assessments and treatment. These findings correspond with an interpretation that authors to a large extent were seeking to get others to recognize a responsible medical practice or a technically advanced hospital activity. However, the activity was often described with a brief introduction and without linking the activity to what will happen later. In this way, the activity is isolated in time and space, and may be perceived as a breach of continuity.

The identities enacted (Tool 5) were often the ones of trustworthy and dedicated clinicians, but in some cases the author may also assume the role of a normative authority.

Texts can affect or alter relationships (Tool 6). The use of specialist language, the focus on technical procedures and investigations and the omission of topics relevant to the follow-up in primary care and future collaboration, may be seen as effectively maintaining a distant relationship between the hospital physician and colleagues in primary health care.

The GP is in the figured worlds (Tool 7) of the authors sometimes situated quite differently from his or her position in real life. The previously mentioned and much used phrase “further follow-up by the GP” implies an assumption that such follow-up would take place, even if no appointment was made and also where it was not clear that the patient had been instructed to book an appointment with his or her RGP. In some cases, a deadline was also set for when a follow-up consultation with the GP should occur. In addition to the assumption that the GP can prioritize such a consultation within the specified deadline, this also implies an assumption that the GP has a way of summoning the patient at relatively short notice.

The discharge summaries did not often invite to a collaboration (Tool 8) and we could not find anything indicating that the doctors at the hospital made themselves available for collaboration on the patient care after discharge. On the other hand, we found that the hospital doctors sometimes delegated tasks to the GPs, asked the GPs to complete some investigation or check deviating findings made at the hospital.

The patient’s voice (Tool 9) was given little priority in most texts. Complaints or description of symptoms on admission were referred to, but wishes, preferences, concerns or thoughts beyond this were usually not mentioned. One discharge summary included the patient’s as well as the closest relatives’ worries about functioning, but this seemed to be an exception.

The recipients (Tool 10) were listed in the heading of the discharge summaries, but authors could also have other readers in mind when writing the texts. Examples of this could be the patients themselves, patients’ relatives, a senior countersigning physician, a lawyer, or some authority.

When we combined the “Recipient Tool” with “The Significance Tool,” it became clear that some recipients stood out as more significant than others. Colleagues in other departments in hospitals were given a higher priority than colleagues in primary care.

## Discussion

Based on the discharge summaries for patients with stroke, this study has identified several obstacles to knowledge transfer and collaboration within the health care services. The absence of post discharge collaboration initiatives expressed in the discharge summaries stands out as a main finding.

The breach of continuity is another main finding. The mapping of guideline recommended content categories in the discharge summaries showed that some forms of content were more often omitted than other forms of content. The material had a clear tendency; matters close to the actual work situation of the author of the discharge summaries are mentioned, while matters more distant are omitted. This is in agreement with the findings and interpretations in the part of the discourse analysis where we applied “The Subject Tool” and found that the topic seemed to be what happened there and then. Possible reasons not to include certain topics could be that they do not seem relevant, that they are regarded to be the responsibility of primary health care, that the specialist opposes the recommendations of the guidelines, or that such topics may generate undesired extra work or responsibility here and now or after discharge.

The fact that the described activities focused on what happened there and then is not necessarily problematic in itself, one of the main functions of any discharge summary must be to communicate what has happened during the stay. The fact that there often was a lack of connection to what was going to happen after discharge, may be more problematic. In this way, the hospital activities are disconnected from the continuous care of the patient. In addition, we found that the hospital doctors were often trying to end their responsibility for the patients and that the patient’s voice most often was absent.

The discharge summary is a text written in one discourse and often, if not always, read in another. Being the only document carrying medical information concerning the individual patient, as the responsibility for treatment and care passes from the hospital to the RGP, it is an essential part of the conversation between the discourses. The health authorities are clear on what themes they want to have included in this conversation. However, our study shows that this conversation is broken. The discharge summaries omit many of the elements that the health authorities have prescribed as important parts of the communication [5].

### Findings in the light of current knowledge

The discharge summaries are tools for communication at a point where the responsibility for the treatment of the patients is handed over from the hospital to the GP. At this point, three stakeholder organizations and their respective discourses meet. The first is the discourse of the health authorities, influenced by current evidence base and scientific insights in the field of stroke research and by national health politics. This discourse is here represented by the normative guideline for treatment and rehabilitation after stroke [5].

The second is the specialized clinical medicine practiced in hospitals. The clinical discourse is also influenced hypotheses about life and death, of ethical choices and of therapeutic decisions [29] but still with a focus on the one illness at hand. Traces of this social practice can be found in the text of the discharge summaries [30].

The third is the discourse of general practice. In contrast to other parts of medicine, where the doctor is concerned with one particular organ or technology, doctors in general practice are to a greater extent concerned with the patient as a person [31, 32]. In other parts of medicine, the doctor-patient relationship most often is of short duration. In general practice, the continuous relationship with the patient is essential, the GP must be pragmatic and the clinical practice can only to a limited extent be based on science [33, 34].

Controversies between these discourses sometimes lead to open confrontations and protests, as in the case of WONCAs (The World Organization of National Colleges, Academies and Academic Associations of General Practitioners/Family Physicians) protest against new and stricter guidelines on treatment of hypertension [35].

Our study revealed that the figured worlds in the discharge summaries sometimes situated the GPs quite differently from their positions in real life and that tasks were delegated to the GPs. Delegation of tasks from hospital doctors to GPs has recently led to controversies in Norway. Some GPs have even expressed that they are expected to do secretarial work for the hospital doctors [36]. It has previously been pointed out that poor communication and poor understanding of each other’s role are barriers of interprofessional collaboration within the health care systems [37]. The finding relates to an area where Vangen and Huxhams theory on collaborative advantage [17] is meant to apply. This general theory on collaboration describes how collaborations can reach *“Collaborative advantage – the synergy that can be created through joint working”* or *“Collaborative inertia - the tendency for collaborative activities to be frustratingly slow to produce output or uncomfortably conflict-ridden”* (p.163). Collaborative situations necessitate a focus on aspects such as collective aims, trust, cultural differences, and knowledge transfer. A lack of focus on these challenges in collaborative situations, makes a collaboration more likely to reach “collaborative inertia” than “collaborative advantages.”

In 2014, Hammad et al. found frequent omissions in adherence to UK national guidance for the content of discharge information [38]. This corresponds well with the findings in our present study on discharge summaries for stroke survivors in Norway. In a previous study on the same cohort, we also found that adherence to the guidelines for follow-up of stoke survivors in general practice is weak [12] and also dependent of the degree of multimorbidity among patients who suffers from stroke [27].

We have identified omissions of guideline recommended content and obstacles to collaboration in the discharge summaries. However, we have no reason to believe that hospital specialists in general are unwilling to collaborate with GPs. On the contrary, available knowledge suggests the opposite. In a Norwegian qualitative study on the referring process [39], Thorsen et al. found that all the interviewed hospital doctors emphasized the importance of good communication and cooperation with GPs. Berendsen et al. found that hospital specialists in The Netherlands were positive to knowledge transfer to GPs as well as to collaboration with GPs [40].

It has previously been pointed out that differences in discourses provide difficulties in aspects of communication between hospital physicians and GPs [41]. Although there is no tradition for employing methods from literacy on patient records, it has been done before [42]. Discourse analytical methods have also been utilized on other material in health services research, e.g. on recorded conversations between health care workers in different settings [43] and on interviews with clinicians [44]. It has, however, been suggested that discourse analysis is an underutilized methodology within health care system research [45], and a search in PubMed performed in September 2019, using the phrase “discharge summary discourse analysis” did not return any results. We were therefore not able to compare the findings in the discourse analysis part of this study with previous findings from the same field.

### Strengths and limitations

To our knowledge, this is the first study involving discourse analysis of discharge summaries for patients with stroke. We examined the discharge summaries, utilizing tools built on perspectives from different approaches and disciplines [22]. A discourse analysis is used to make claims about for example written texts, such as discharge summaries, based on interpretations. Transdisciplinary convergence is proposed to validate discourse analysis approaches to research. When interpretations based on the use of tools of analysis that go beyond one discipline converge, claims of validity can be made [46]. We claim this was the case in our present study.

One could argue that another selection of tools may have led to other conclusions. This is a consequence of the discourse analysis as a method. Nevertheless, the tools applied are acknowledged tools of discourse analysis, they are available to the reader, and as far as we could see in the process, they were the best tools available to provide information on the topics we were exploring in this study.

It may be argued that the perspective of the discharging physician is only represented in the discharge summary and not through for example interviews or focus groups. Although the aim of this study has been to assess the discharge summaries and not the authors’ perspectives, research on the perspectives of both the authors and recipients of the discharge summaries could contribute to a better understanding of the communication between the various parts of the health care system.

The material in this study consists of hospital discharge summaries for patients discharged in 2011 and 2012. It may be considered a weakness that discharge workflow and discharge summaries may have evolved since then, and that lack of knowledge transfer and collaboration initiatives may be less common in different settings, in other hospitals and in other countries. However, the findings in this study are reported in the context of the guideline recommendations for the discharge summaries at the time of study. More research on the discharge summaries may broaden the empirical basis and provide more nuance in the understanding of the communication between hospitals and primary care.

The situated position as reader, analyst and interpreter is continually changing during the process of this project. This means that the researchers probably will emphasize other things at the beginning of the project than at the end of the project. Different readers of the summaries will represent different situated positions that will lead to different interpretations of the text [47]. Despite these weaknesses, we claim that the findings are valid for the discharge summaries in this study.

## Conclusions

This study has shown that the discharge summaries for stroke survivors residing in the communities did not include all the content recommended by the Norwegian national guideline for the treatment and rehabilitation in stroke. The discharge summaries have the potential to serve as tools for collaboration across boundaries within the health care services. They can also be utilized for knowledge transfer, and guideline implementation in general practice. This study, however, pointed out that the discharge summaries were not optimized for such purposes. The discharge summaries focused the fragments of the health service provided by the hospital. In this process, they also disconnected the hospital, its doctors and other groups of health care professionals from the continuous care for the patients. By doing this, they may contribute in sustaining the gap between the discourse in hospital medicine and the discourse in general practice.

Health services are not a seamless continuum, they are still fragmented. One explanation lies in the broken conversation between the different discourses in research, hospital medicine and primary health care. The guidelines express ambitions of collaboration and continuous chains of care across boundaries between secondary and primary health care services. These ambitions were not reflected in the discharge summaries for stroke survivors. We believe that further research on the perspectives of the different stakeholders in this collaboration is necessary to identify ways of bridging the gaps between the discourses involved and contribute to continuous health care services.

## Supplementary Information

**Additional file 1.**

## Data Availability

The datasets used and/or analysed during the current study are available from the corresponding author on reasonable request.

## Abbreviations

GP: General Practitioner
Helfo: The Norwegian Health Economics Administration
ICD 10: International Classification of Diseases and Related Health Problems, 10th revision
ICPC-2: International classification of primary care, 2nd revision.
RGP: Regular general practitioner
WHO: World Health Organization
Wonca: World Organization of Family Doctors
