# Supplementary material for: Stroke follow-up in primary care: a discourse study on the discharge summary as a tool for knowledge transfer and collaboration
Source: BMC Health Serv Res. 2021 Jan 7;21:41. doi: 10.1186/s12913-020-06021-8 (PMC7792345; doi:10.1186/s12913-020-06021-8)
Supplement: Supplementary file 1 — Additional file 1. [file 12913_2020_6021_MOESM1_ESM.docx]

**Operational definitions list:**

1. *The kind of stroke and its localization in the brain:* Count discharge summaries that include a description of the kind of stroke *and* its location in the brain. The localization of the stroke is provided when either clinically or radiologically described. Count also when not applicable, e.g. a new admission for the same stroke.
2. *The cause of the stroke:* Count discharge summaries that provide a description of the cause of the stroke, count also discharge summaries that provide an assessment of risk factors. Count also when not applicable (see also 1)).
3. *A short description of the treatment and the diagnostic investigation*: Count discharge summaries with any description of the treatment *and* the diagnostic investigation.
4. *Complications (if applicable):* Refer to page 48 of the National Guideline for stroke [1]. Count discharge summaries with new diagnosis of: Seizures, Raised Intracranial Pressure, Infections, Venous thrombosis, Cardial arrythmia, Risk of falls, Pain, Psychiatric symptoms, Stress Ulcer or any Gastrointestinal bleeding, or Dysphagia.
5. *The patients` level of function on discharge:* Count discharge summaries that provide any information on the patient´s level of function on discharge.
6. *Prognosis, including prognosis for driver`s license and work:* Count discharge summaries that provide a prognosis *including* prognosis for driver`s license and work. Count discharge summaries also when driver`s license and work is not applicable.
7. *Assessment of the necessity for further diagnostic investigations:* Count discharge summaries with any assessment of the necessity for further diagnostic investigations
8. *Medication at discharge:* Count discharge summaries that provide an overview of medication at discharge.
9. *Further treatment and treatment goals for the blood pressure and blood lipid values:* Perform separate counts: I) Count discharge summaries that provide treatment goals for blood pressure and II) Count discharge summaries that provide treatment goals for blood lipid values.
10. *Plans for the follow-up:* Count discharge summaries that provide any plan for the follow-up in general practice.

1. The Norwegian Directorate of Health. Nasjonal faglig retningslinje for behandling og rehabilitering ved hjerneslag [National guideline for treatment and rehabilitation in stroke] (In Norwegian)*.* Oslo: The Norwegian Directorate of Health (Helsedirektoratet); 2010.
